# Effectiveness of the ColorApp Mobile App for Health Education and Promotion for Colorectal Cancer: Quasi-Experimental Study

**DOI:** 10.2196/15487

**Published:** 2020-02-25

**Authors:** Nor Azwany Yaacob, Muhamad Fadhil Mohamad Marzuki, Najib Majdi Yaacob, Shahrul Bariyah Ahmad, Muhammad Radzi Abu Hassan

**Affiliations:** 1 Department of Community Medicine School of Medical Sciences Universiti Sains Malaysia Health Campus Kubang Kerian, Kelantan Malaysia; 2 Unit of Biostatistics and Research Methodology School of Medical Sciences Universiti Sains Malaysia Health Campus Kubang Kerian Malaysia; 3 Non-Communicable Disease Control Unit Kedah State Health Department Alor Setar, Kedah Malaysia; 4 Clinical Research Centre Hospital Sultanah Bahiyah Alor Setar, Kedah Malaysia

**Keywords:** colorectal cancer, mobile app, effectiveness, knowledge, attitude

## Abstract

**Background:**

Lack of knowledge and poor attitude are barriers to colorectal cancer screening participation. Printed material, such as pamphlets and posters, have been the main approach in health education on disease prevention in Malaysia. Current information technology advancements have led to an increasing trend of the public reading from websites and mobile apps using their mobile phones. Thus, health information dissemination should also be diverted to websites and mobile apps. Increasing knowledge and awareness could increase screening participation and prevent late detection of diseases such as colorectal cancer.

**Objective:**

This study aimed to assess the effectiveness of the ColorApp mobile app in improving the knowledge and attitude on colorectal cancer among users aged 50 years and older, who are the population at risk for the disease in Kedah.

**Methods:**

A quasi-experimental study was conducted with 100 participants in Kedah, Malaysia. Participants from five randomly selected community empowerment programs in Kota Setar district were in the intervention group; Kuala Muda district was the control group. Participants were given a self-administered validated questionnaire on knowledge and attitudes toward colorectal cancer. A mobile app, ColorApp (Colorectal Cancer Application), was developed as a new educational tool for colorectal cancer prevention. The intervention group used the app for two weeks. The same questionnaire was redistributed to both groups after two weeks. The mean percentage scores for knowledge and attitude between groups were compared using repeated measure ANCOVA.

**Results:**

There was no significant difference in age, sex, highest education level, current occupation, and diabetic status between the two groups. The number of smokers was significantly higher in the intervention group compared with the control group and was controlled for during analysis. The intervention group showed a significantly higher mean knowledge score compared with the control group with regards to time (Huynh-Feldt: *F*_1,95_=19.81, *P*<.001). However, there was no significant difference in mean attitude scores between the intervention and control groups with regards to time (*F*_1,95_=0.36, *P*=.55).

**Conclusions:**

The ColorApp mobile app may be an adjunct approach in educating the public on colorectal cancer.

## Introduction

There were 1.7 million new cases of colorectal cancer in 2015, with 832,000 deaths globally [1]. It is the third most commonly diagnosed type of cancer. However, colorectal cancer was reported by the National Cancer Registry as the second most common cancer in Malaysia between 2007 and 2011. The total number of cancer cases registered was 13,693 or 13.2% of all cancer types. The age-standardized rate of colorectal cancer was 14.6 per 100,000 population for males and 11.1 per 100,000 population for females [2]. There is a wide disparity in the country-specific incidence in Southeast Asia, with the highest incidence reported in Singapore (age-standardized rate 33.7 per 100,000 population) [3]. However, trends for both the incidence and the mortality rates for Singapore have been stable and even declining since 2000. Other countries, such as Indonesia, Thailand, Vietnam, and Myanmar, have comparatively lower incidence rates of colorectal cancer than Malaysia [4].

People living in Malaysia, Singapore, India, and Brunei were reported to have a significantly more negative response toward and lack of intention to undergo colorectal cancer screening compared with people in the Philippines and Japan. The public’s low knowledge and awareness, as well as their poor attitude toward screening, may explain the negative perception and lead to a low rate of screening [5]. Those at high risk of getting colorectal cancer were also reported to have low intention; only 38% of them were willing to be screened [6]. Furthermore, the intention to go for screening among people aged 50 years and older is the lowest compared with younger age groups. Lack of knowledge or education is the most critical barrier reported in many studies [5,7-11]. Poor knowledge by the general public about the risk factors, signs, symptoms, and screening tests available leads to low awareness of the importance of preventive action including taking a screening test. In addition, background education level plays an important role in determining health literacy, especially in rural areas. Health education and promotion on colorectal cancer are still not as rigorous as the lung, breast, and cervical cancer prevention activities in Malaysia despite increasing incidence from year to year [11]. Therefore, the awareness program for colorectal cancer and health promotion and education need to be strengthened in Malaysia.

Mobile phone usage is on the rise leading to high demand for mobile phone apps [12]. These apps could be used for social interaction, education, entertainment, as well as personal health. Mobile apps have been shown to have a potential role in health awareness and behavioral changes [13,14]. The mobile phone has been studied as a platform for intervention programs, such as text messaging interventions for smoking cessation [15,16], for diabetic education [17], to monitor patient health status [18], to increase physical activity [13], and for dietary management [19]. The integration of multiple functions of a mobile phone with a mobile app enables not only health education but also the monitoring of user progress, providing health reminders, and sharing outcomes with health care providers or friends to maintain user motivation. A mobile app also provides a useful and faster way to disseminate information, such as risk factors and preventive measures, at a low cost [12]. By 2010, there were more than 7000 apps developed for health education and promotion purposes [20].

A mobile app named ColorApp (Colorectal Cancer Application), intended for the population at risk in Malaysia, was developed as a new tool for health education and promotion on colorectal cancer [21]. Its usability has been shown in a usability study published previously [21]. However, we do not know whether this usable mobile app can improve the knowledge and attitudes of users on colorectal cancer. Therefore, this study aimed to assess the effectiveness of the ColorApp mobile app in improving the knowledge and attitudes on colorectal cancer among users aged 50 years and older who are the population at risk for the disease in Kedah.

## Methods

### Overview

A quasi-experimental study was conducted in the state of Kedah, Malaysia. The two most populous districts with good broadband coverage were selected. These districts have active community empowerment programs at different localities. This community empowerment program, known by the acronym KOSPEN, aimed to empower people to achieve better health by reducing the behavioral and modifiable noncommunicable disease risk factors [22]. Kota Setar district was chosen as the intervention district and Kuala Muda district as the control district. Five KOSPEN localities in each district were randomly selected. Ten eligible program participants were randomly selected from each locality to achieve a total of 100 participants, 50 in each group. Eligible participants were those aged 50 years and older, who owned an Android mobile phone and had never been diagnosed with any type of cancer. This study was approved by the National Medical Research Registry, Malaysia (NMRR-17-2623-38675 [IIR]) and Human Research Ethics Committee USM, Malaysia (USM/JEPeM/17110601).

There were two tools—one apps and one questionnaire—used in this research study, which are described subsequently.

### ColorApp Mobile App

ColorApp is a newly developed mobile app for health promotion and education on colorectal cancer. It consists of 10 interfaces on introduction to colorectal cancer, signs and symptoms, risk factors, preventions, screening programs in Malaysia, and immunochemical fecal occult blood test kits. It also has two interactive pages to stratify the user’s risk of disease and a health calculator that provides a recommendation on the need for screening as well as ideal body weight, recommended blood pressure, glucose, and cholesterol level. A video on colorectal cancer is also available. ColorApp has been tested and proven to be usable by the intended users. The development process and usability study have been published recently [21].

### Self-Administrated Questionnaire on Knowledge and Attitude on Colorectal Cancer

This is a validated Malay version of a self-administered questionnaire that consists of four sections: sociodemographic information, knowledge of the general aspects of colorectal cancer, attitude on colorectal cancer, and practices of lifestyle and colorectal cancer screening [23]. Due to time and logistic constraints, this study only assessed the immediate changes, which were knowledge and attitude on colorectal cancer. Practice change on colorectal cancer screening required a longer duration of follow-up; thus, it was not possible to be covered during the study period. The number of items for knowledge and attitude were 29 and 10, respectively. A 5-point Likert scale was used: strongly agree, agree, neutral, disagree, and strongly disagree. There were positive and negative items; scores of 5 to 1 were used for positive items and reverse scores for negative items. Summation of the total score for each domain was converted into a percentage score. The mean percentage score of knowledge and attitude before and after using ColorApp were computed and compared. The Cronbach alphas for knowledge and attitude domain were .65 and .82, respectively. The self-administered questionnaire on knowledge and attitude on colorectal cancer is attached in Multimedia Appendix 1.

### Data Collection Method

Participants were invited to participate in this study through our program coordinator. During the preintervention period, participants were invited to a community health program at selected localities. A health talk on local public health concerns was conducted to gather all the participants and as an add-on benefit for all. The participants were then given a validated self-administered questionnaire on colorectal cancer knowledge and attitude. On completion of the questionnaire, participants from the intervention group were introduced to ColorApp and asked to install it onto their mobile phone. The ColorApp prototype was published in the Google Play store as a beta option; only users who were provided with the app link were able to install it to avoid cross-contamination (ie, users using the app that did not sign up as participants in our study). Participants in the intervention group were instructed to use ColorApp for two weeks at their own convenience. No mobile app was introduced to the control group. The same questionnaire was readministered after two weeks of using ColorApp during a second community health activity at each locality. For the benefit of the participants, they were given a health talk on colorectal cancer. The control group participants were also introduced to ColorApp after all participants submitted the postintervention questionnaire. The mean percentage scores for knowledge and attitude between groups were compared using repeated measures ANCOVA (analysis of covariance) because it adjusted for the age and smoking status of participants. Age was controlled during the study design, and smoking status was controlled because it showed a significant difference between the intervention and control groups.

## Results

### Overview

There was no difference in terms of age, sex, highest education level, and current occupation between the control and intervention groups. However, there was a significant difference in smoking status; more participants from the intervention group smoked compared with the control group (Table 1).

Comparisons of the mean percentage scores of knowledge and attitude on colorectal cancer between the intervention and control groups with consideration of time pre- and postusage of the mobile app comprised three separate results.

**Table 1:** Sociodemographic and health characteristics of participants by intervention and control group (N=100).

Variables		Intervention (n=50)	Control (n=50)	*P* value
Age (years), mean (SD)		56.0 (5.69)^a^	55.8 (4.76)^a^	.86^a^
**Sex, n (%)**				
	Male	25 (50)	25 (50)	>.99^b^
	Female	25 (50)	25 (50)	
**Highest education, n (%)**				
	Tertiary	7 (14)	3 (6)	.40^b^
	Secondary	33 (66)	35 (70)	
	Primary	10 (20)	12 (24)	
**Current occupation, n (%)**				
	Employed	17 (34)	22 (44)	.31^b^
	Unemployed	33 (66)	28 (56)	
**Smoking status, n (%)**				
	Yes	17 (34)	7 (14)	.02^b^
	No	33 (66)	43 (86)	
**Diabetes status, n (%)**				
	Yes	10 (20)	6 (12)	.28^b^
	No	40 (80)	44 (88)	
**Body mass index category, n (%)**				
	Underweight	3 (6)	1 (2)	.61^c^
	Ideal body weight	14 (28)	18 (36)	
	Overweight	19 (38)	20 (40)	
	Obese	14 (28)	11 (22)	

^a^Independent *t* test.

^b^Chi-square test.

^c^Fisher exact test.

### Within-Group Difference (Time Effect)

The time-effect mixed-design repeated measures ANCOVA analysis showed that there was a significant increment in knowledge scores among participants in the intervention group after using the mobile app (Wilks’ lambda, *F*_1,47_=7.38, *P*=.007). Participants in the intervention group had significantly better knowledge postintervention compared with preintervention. Within the control group, knowledge scores were observed to be lower at postintervention; however, it was not statistically significant (Wilks’ lambda, *F*_1,47_=0.71, *P*=.40).

The time-effect mixed-design repeated measures ANCOVA analysis for attitude showed that there was no significant change of mean attitude score after using the mobile app. The mean percentage score difference in attitude before and after ColorApp usage was not significant for the intervention group (Wilks’ lambda, *F*_1,47_=0.28, *P*=.60) or the control group (Wilks’ lambda, *F*_1,47_=1.68, *P*=.20). Participants’ attitudes from both groups did not change significantly postintervention.

The comparison of adjusted mean percentage scores of knowledge and attitude within the intervention group are shown in Table 2.

**Table 2:** Comparison of adjusted mean percentage scores of knowledge and attitude within the intervention and control group (n=50).^a^

Time	Intervention group		Control group	
	Adjusted mean difference (95% CI)	*P* value	Adjusted mean difference (95% CI)	*P* value
Postintervention knowledge score−preintervention knowledge score	2.65 (0.75, 4.55)	.007	0.66 (−0.91, 2.23)	.40
Postintervention attitude score−preintervention attitude score	0.77 (−2.17, 3.70)	.60	−3.00 (−7.65, 1.65)	.20

^a^Two-way mixed-design repeated measure ANCOVA was applied and adjusted for smoking.

### Between-Group Difference (Intervention Effect)

The intervention-effect mixed-design repeated measures ANCOVA analysis showed that there was an overall significant difference in mean percentage score of knowledge between intervention and control groups regardless of time (*F*_1,95_=11.36, *P*=.001). For attitude, there was no overall significant difference between the intervention and control groups on the mean percentage score of attitude regardless of time (*F*_1,95_=0.42, *P*=.52). The adjusted mean difference between intervention and control for percentage score knowledge was 3.34 (95% CI 1.37-5.31, *P*=.001) and for attitude was −0.784 (95% CI −3.18 to 1.62, *P*=.34).

### Between-Group Difference Based on Time (Interaction Effect)

**Figure 1:**
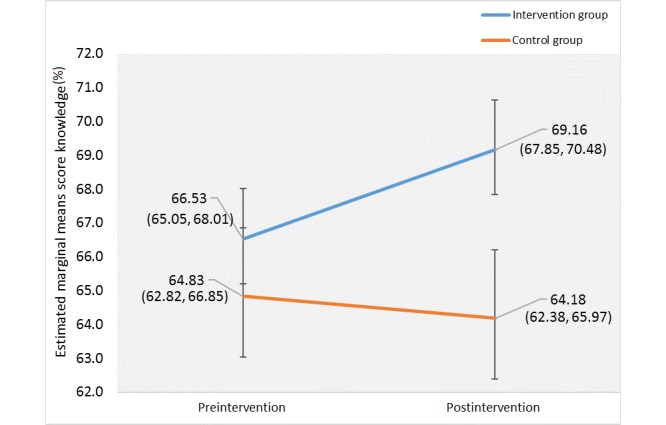
Profile plot of the estimated marginal means of the mean percentage scores for knowledge between pre- and postusage of ColorApp.

Two-way mixed-design repeated measures ANCOVA was applied and adjusted for age and smoking status. There was no mean percentage score difference between groups during preintervention (Huynh-Feldt: *F*_1,95_=1.82, *P*=.21); however, there was a significant difference between groups during postintervention (Huynh-Feldt: *F*_1,95_=19.81, *P*<.001; partial eta squared=0.610). There was a significant difference in mean percentage score knowledge between the intervention and control groups with regards to time (Wilk’s lambda, *F*_1,95_=6.20, *P*=.02). The interaction effect produced a significant difference in mean percentage score knowledge with consideration of time between pre and post usage of ColorApp (Figure 1).

**Figure 2:**
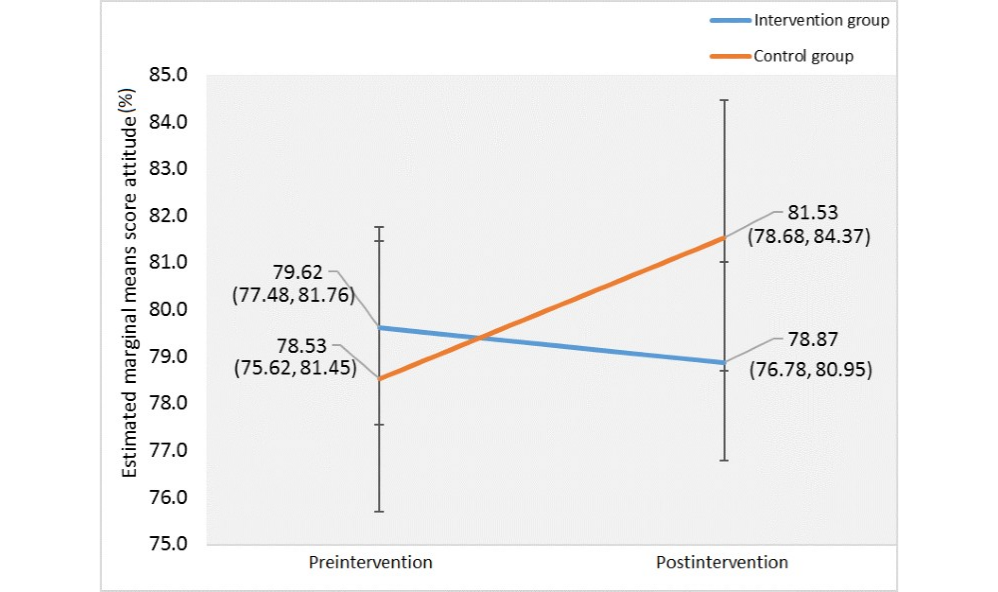
Profile plot of the estimated marginal means of the mean percentage scores for attitude between pre- and postusage of ColorApp.

For attitude, there was no difference between groups during preintervention (Huynh-Feldt: *F*_1,95_=0.36, *P*=.55) and postintervention (Huynh-Feldt: *F*_1,95_=2.24, *P*=.14). There was also no significant difference in mean percentage score attitude between the intervention and control groups with regard to time (Wilks’ lambda, *F*_1,95_=1.98, *P*=.16). The interaction effect produced no significant difference in the mean percentage score attitude with consideration of time between pre- and postusage of ColorApp (Figure 2).

Based on the analysis, the intervention group showed a significant improvement in their knowledge score after using ColorApp, but not their attitude score. The partial eta squared for intervention effect was 0.610, indicating a large effect size based on Cohen’s guidelines [24].

## Discussion

This study shows an improvement in knowledge of colorectal cancer after the use of the ColorApp app, but it did not affect attitude toward preventing colorectal cancer. ColorApp was developed to be used by people aged 50 years and older who are at higher risk of getting colorectal cancer. It was designed to improve their general knowledge of colorectal cancer and attitudes toward their disease susceptibility, disease prevention through a healthier lifestyle, and taking a screening test. A quasi-experimental study was conducted because this was a population-based study in which it was not feasible to randomize at the individual level without contaminating the control group [25]. Two different districts were used to avoid cross-contamination (participants in the control group were unable to download and use the mobile app). However, districts (or geographical regions) with similar population characteristics were chosen to ensure comparability between groups, as shown by the baseline participants’ characteristics. Factors such as age, gender, educational level, and occupation, which may influence the level of knowledge and attitudes on colorectal cancer, were similar at baseline. The smoking habit was significantly different between the intervention and control groups and was controlled during the multivariate analysis.

The time-intervention effect showed a significant improvement of overall knowledge on colorectal cancer among participants in the intervention group compared with the control group. This indicates that ColorApp can be a tool to deliver health information on colorectal cancer even to older users. This app provides information on the epidemiology of colorectal cancer in Malaysia, signs and symptoms, risk factors, prevention, and colorectal cancer screening methods, as described in detail in the development publication [21]. Many studies have supported the use of mobile phones in improving the user’s or patient’s knowledge and awareness of the disease. A systematic review of health care apps for mobile phones found more than 15 apps that focused on disease management for chronic illness were able to improve patients’ knowledge. Thus, mobile apps are able to play an important role in disseminating evidence-based health information and are a tool for disease self-management, remote monitoring, and mobile clinical communication [26]. A study in China suggested that the use of a mobile app could improve the user’s experience, especially on the accessibility to health information, leading to positive health outcomes [27]. In another study involving diabetic patients in England, the majority of participants agreed that a mobile health app had great potential in health promotion and was beneficial and helpful for them to live a healthier lifestyle [28]. Therefore, increasing mobile phone ownership should be fully used in delivering health education and promotion to intended users. It can be a good complement to other modalities of health education to enhance the effect of education to change people’s behavior.

However, this study found no significant improvement in the overall attitude of participants on the prevention of colorectal cancer in the intervention group compared with the control group. The participants in the intervention group were given only two weeks to use the ColorApp app before conducting the postintervention assessment. The duration may have been inadequate to assess attitude change on colorectal cancer. Changing attitudes toward perceived susceptibility to the disease and the benefits of taking preventive action (eg, choosing a healthy lifestyle) and health-seeking behavior (eg, taking a stool screening test) is not as easy as improving people’s knowledge. Attitude change also requires consistent and congruent information that needs to be delivered through various information dissemination methods and includes messages that are high in affect or emotion and connect attitudes to past behaviors [29].

Changing attitudes is even more challenging when the health education messages contradict the user’s beliefs, ethics, and cultural values [30]. By going through all pages of the app, reading the information available, and using the interactive function, users may gain more knowledge about colorectal cancer. Early detection of cancer, even by means of a noninvasive procedure, can also be an unpleasant life experience. Changing attitudes toward a healthier lifestyle, such as eating more vegetables, being physically active, and stopping smoking, may take longer because they involve their personal value(s).

Although this study was unable to show a significant change in attitude toward colorectal screening, this should not be the reason for not using mobile apps as a means of health promotion and education because other studies have proved an association between knowledge of colorectal cancer and colorectal cancer screening rates [31-33]. A qualitative study was conducted with a focus group of 55 African Americans aged 50 years and older, which found that lack of knowledge is one of the major barriers to screening [31]. A similar finding was seen in other studies, such as a study involving 247 participants in New Mexico during a community event [32] and another study involving 1060 randomly selected visitors at Razavi Hospital of Mashhad, Iran [33]. Looking at the local context, studies have shown that knowledge and awareness of colorectal cancer among the Malaysian population is still low [5,34,35]. Therefore, any means that may improve the knowledge of the population on colorectal cancer should be fully utilized to increase screening participation. However, a longer period of study intervention and follow-up may provide better evidence for the effectiveness of an educational intervention.

In conclusion, the advancements in communication technology should be fully used by health care providers in disseminating health education messages to the target population. A mobile app such as ColorApp is a way forward for health promotion and education, particularly in the prevention and early detection of colorectal cancer. ColorApp is currently published in version 14. It can be downloaded for free from the Google Play store.

## Appendix

Multimedia Appendix 1 Self-administered questionnaire on knowledge and attitude on colorectal cancer.

## Abbreviations

ANCOVA: analysis of covariance
