# Supplementary material for: Effectiveness of the ColorApp Mobile App for Health Education and Promotion for Colorectal Cancer: Quasi-Experimental Study
Source: JMIR Hum Factors. 2020 Feb 25;7(1):e15487. doi: 10.2196/15487 (PMC7064964; doi:10.2196/15487)
Supplement: Multimedia Appendix 1 [file humanfactors_v7i1e15487_app1.doc]

Self-Administered Questionnaire on Knowledge and Attitude on Colorectal Cancer

1 2 3 4 5

Very Disagree Very Agree

| Question on Knowledge | | 1 | 2 | 3 | 4 | 5 |
| --- | --- | --- | --- | --- | --- | --- |
| *K1. | Kanser kolorektal ialah kanser yang paling kerap berlaku di Malaysia.  *(Colorectal cancer is the most common cancer in Malaysia.)* |  |  |  |  |  |
| K2. | Kanser kolorektal lebih kerap berlaku pada mereka yang berusia lebih daripada 50 tahun.  *(Colorectal cancer is more common in people over the age of 50 years.)* |  |  |  |  |  |
| *K3. | Kanser kolorektal lebih kerap berlaku di kalangan wanita.  *(Colorectal cancer is more common in women.)* |  |  |  |  |  |
| Pada pandangan saya, berikut ialah faktor risiko/penyebab kanser kolorektal:  *(In my view, here are the risk factors / causes of colorectal cancer:)* | | | | | | |
| *K4a. | Berkongsi makanan  *(Sharing food)* |  |  |  |  |  |
| *K4b. | Kesan daripada buasir  *(Effects of hemorrhoids)* |  |  |  |  |  |
| *K4c. | Kesan jangkitan kuman  *(Effects of bacterial infection)* |  |  |  |  |  |
| K4d. | Banyak pengambilan makanan bersalai/bakar dalam hidangan harian  *(High intake of smoked/grilled food in daily diet)* |  |  |  |  |  |
| K4e. | Baka daripada ibubapa (keturunan)  *(Inherited from parent (hereditary))* |  |  |  |  |  |
| K4f. | Daripada ketumbuhan (polip) di dalam usus  *(From tumors (polyps) in the intestine)* |  |  |  |  |  |
| *K4g. | Akibat daripada sentuhan dengan pengidap kanser kolorektal  *(As a result of direct contact with colorectal cancer patient)* |  |  |  |  |  |
| Berikut adalah tanda-tanda kanser kolorektal:  *(Here are some signs and symptoms of colorectal cancer:)* | | | | | | |
| *K5a. | Buasir  *(Hemorrhoids)* |  |  |  |  |  |
| K5b. | Bengkak di dalam perut  *(Swelling in the abdomen)* |  |  |  |  |  |
| K5c. | Hilang berat badan  *(Weight loss)* |  |  |  |  |  |
| K5d. | Hilang selera makan  *(Loss of appetite)* |  |  |  |  |  |
| K5e. | Pendarahan semasa membuang air besar  *(Bleeding during bowel movement)* |  |  |  |  |  |
| K5f. | Sembelit yang diikuti cirit-birit  *(Altered bowel movement)* |  |  |  |  |  |
| *K5g. | Susah menelan  *(Difficulty in swallowing)* |  |  |  |  |  |
| K5h. | Rasa tidak puas selepas membuang air besar  *(feeling that the bowel does not empty completely)* |  |  |  |  |  |
| *K5i. | Perut rasa pedih  *(Stomach pain)* |  |  |  |  |  |
| K6. | Kanser kolorektal mempunyai peluang untuk sembuh sekiranya dikesan lebih awal.  *(Colorectal cancer has a chance to heal if it is detected earlier)* |  |  |  |  |  |
| K7. | Kanser kolorektal boleh dirawat secara pembedahan.  *(Colorectal cancer can be treated surgically.)* |  |  |  |  |  |
| *K8. | Kanser kolorektal boleh diubati dengan efektif secara radioterapi sahaja.  *(Colorectal cancer can be treated effectively only by radiotherapy)* |  |  |  |  |  |
| K9. | Ujian najis untuk saringan kanser kolorektal boleh didapati di klinik kesihatan.  *(The stool test for colorectal cancer screening is available in health clinics)* |  |  |  |  |  |
| *K10. | Ujian saringan najis disyorkan kepada golongan yang berumur 30 tahun dan ke atas.  *(The screening test is recommended for people aged 30 years and above)* |  |  |  |  |  |
| *K11. | Ujian saringan disyorkan bagi mereka yang mempunyai ahli keluarga yang mengidap kanser kolorektal sahaja.  *(Screening tests are recommended for those with family members who have colorectal cancer only.)* |  |  |  |  |  |
| K12. | Pengambilan tisu melalui penyuluhan usus (kolonoskopi) diperlukan untuk mengesahkan kanser kolorektal jika ujian saringan didapati positif.  *(A sampling of tissue via* *colonoscopy is required to diagnose colorectal cancer if the screening test is positive)* |  |  |  |  |  |
| Kanser kolorektal boleh dikesan melalui:  *(Colorectal cancer can be detected through:)* | | | | | | |
| K13a. | Ujian darah dalam najis (FOBT)  *(Blood test in stool (FOBT))* |  |  |  |  |  |
| K13b. | Kolonoskopi (suluh usus)  *(Colonoscopy)* |  |  |  |  |  |
| *K13c. | Ultrasound  *(Ultrasound)* |  |  |  |  |  |

1 2 3 4 5

Very Disagree Very Agree

| Question on Attitude | | 1 | 2 | 3 | 4 | 5 |
| --- | --- | --- | --- | --- | --- | --- |
| A1. | Saya berisiko mendapat kanser kolorektal.  *(I am at risk of getting colorectal cancer)* |  |  |  |  |  |
| A2. | Saya percaya kanser kolorektal boleh dicegah.  *(I believe colorectal cancer can be prevented)* |  |  |  |  |  |
| *A3. | Saya percaya perkhidmatan perubatan tradisional mampu merawat kanser kolorektal.  *(I believe traditional medical services are able to treat colorectal cancer)* |  |  |  |  |  |
| A4. | Saya sanggup memperbanyakkan pengambilan sayur-sayuran untuk mengelakkan diri daripada kanser kolorektal.  *(I am willing to increase the consumption of vegetables to avoid colorectal cancer)* |  |  |  |  |  |
| A5. | Saya sanggup melakukan senaman secara berkala untuk mengelakkan diri daripada kanser kolorektal.  *(I'm willing to do regular exercise to avoid colorectal cancer)* |  |  |  |  |  |
| A6. | Saya berminat mendapatkan maklumat berkaitan kanser kolorektal.  *(I am interested in getting information on colorectal cancer)* |  |  |  |  |  |
| A7. | Saya akan meluangkan masa untuk mendapatkan ujian saringan dalam usaha mencegah kanser kolorektal.  *(I will take the time to get a screening test in order to prevent colorectal cancer)* |  |  |  |  |  |
| A8. | Saya percaya ujian saringan memberi kebaikan dalam penjagaan kesihatan saya.  *(I believe the screening tests are beneficial in my health care)* |  |  |  |  |  |
| *A9. | Saya tidak perlu melakukan ujian saringan/pengesanan kanser kolorektal kerana saya tidak mempunyai apa-apa tanda dan gejala.  *(I do not need screening / colorectal cancer detection because I do not have any signs and symptoms)* |  |  |  |  |  |
| A10. | Saya percaya Vitamin E boleh mencegah kanser kolorektal.  *(I believe Vitamin E can prevent colorectal cancer)* |  |  |  |  |  |
